# Disruption of global brain network topology in amnestic MCI: evidence from multimodal DTI and fMRI

**DOI:** 10.3389/fnins.2025.1675610

**Published:** 2025-11-25

**Authors:** Yingmei Han, Xue Bai, Weiqing Li, Ze Feng, Bingyuan Chu, Ming Yang, Qingqing Shang, Hanxi Zhang, Xinlu Li, Feng Wang

**Affiliations:** 1 Graduate School of Heilongjiang University of Chinese Medicine, Harbin, China; 2 Division of CT and MRI, First Affiliated Hospital of Heilongjiang University of Chinese Medicine, Harbin, China

**Keywords:** amnestic mild cognitive impairment, global topological properties, brain network, Diffusion tensor imaging, functional magnetic resonance imaging

## Abstract

**Objective:**

This study aims to utilize multimodal neuroimaging techniques to simultaneously analyze global topological properties of white matter structural networks and resting-state functional networks in aMCI patients, comparing them with healthy controls. By conducting independent and integrative analyses of topological impairments in both networks, we seek to systematically characterize the multimodal network disruption patterns in aMCI.

**Methods:**

45 aMCI patients and 42 healthy adults from the First Affiliated Hospital of Heilongjiang University of Chinese Medicine in Harbin, Heilongjiang Province, China, were enrolled. A case-control cross-sectional study was conducted. DTI and rs-fMRI data were collected for all participants. Global topological properties of structural and functional networks were constructed using PANDA and dpabi software and were calculated via graph-theoretical analysis in GRETNA software, followed by statistical comparisons between groups.

**Results:**

In patients with aMCI, the small-world (C_*p*_, aC_*p*_, Lambda, aLambda) of the WM structural network were significantly higher than those in the HC group; Rich-club nodes showed redistribution, and the Rich-club coefficient was decreased; aE_*loc*_ was significantly increased; the Assortativity index (*r* < 0) indicated disassortativity; the Hierarchy index (*b* > 0) exhibited a significant decrease in *b* within the sparsity range of 0.39∼0.4; the synchronization coefficient (*s*) was significantly reduced at sparsity levels ranging from 0.28 to 0.30. For the functional network, the small-world index aL_*p*_ in the aMCI group was significantly lower than that in the HC group; Rich-club nodes showed redistribution, and the Rich-club coefficient was increased within a certain Degree range; aE_*g*_ was significantly increased; the Assortativity index (*r* > 0) indicated assortativity; the Hierarchy index (*b* > 0) was observed within a specific sparsity range.

**Conclusion:**

We identified a “structure-function dissociation” in aMCI, where the structural network suffers from fragmentation and hub disruption, while the functional network compensates through rigid, hyper-localized reorganization with elevated local efficiency. This divergence reveals a core pathological mechanism of the disease.

## Graphical Abstract

**Figure:**
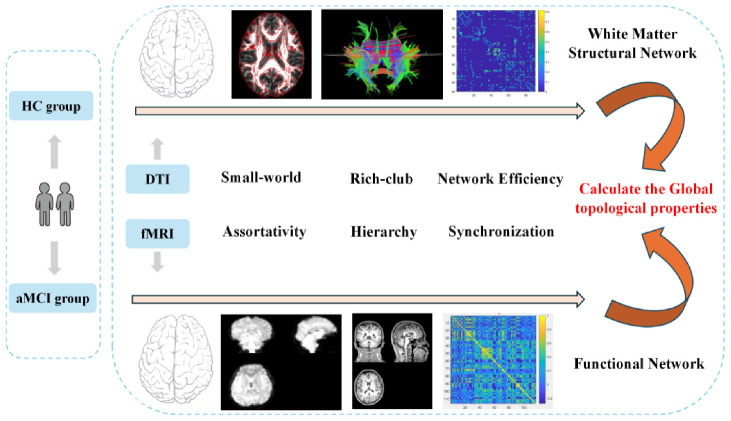
This study employed multimodal neuroimaging (DTI and fMRI) to analyze and compare the global topological properties of both white matter structural networks and functional networks in healthy controls and patients with amnestic mild cognitive impairment (aMCI), thereby revealing potential neural mechanisms underlying aMCI. HC, healthy control; aMCI, amnestic mild cognitive impairment; DTI, diffusion tensor imaging; fMRI, functional magnetic resonance imaging.

## Highlights

- Previous studies have observed differences in the topological properties of WM structural networks or functional networks in MCI compared with healthy controls, subjective cognitive decline, or Alzheimer’s disease dementia. This study specifically focuses on the changes in brain network topological properties of aMCI subtype.
- This study combines DTI and fMRI techniques to analyze the global topological properties of WM structural networks and functional networks in aMCI, which has not been explored in previous research. Furthermore, the potential limitation of heterogeneous sampling sources was mitigated in this study.
- Previous studies have paid relatively less attention to Assortativity, Hierarchy, and Synchronization indices. Based on other common research indices, this study focuses on these three indices to investigate whether the global principles followed by brain networks in healthy individuals and aMCI patients have changed.

## 1 Introduction

Mild cognitive impairment (MCI) represents an intermediate clinical stage between normal aging and dementia, characterized by a significant decline in cognitive abilities that exceeds typical age-related changes yet does not meet the criteria for dementia (Anand and Schoo, 2024; Jia et al., 2020). Clinically, MCI is categorized into amnestic MCI (aMCI) and non-amnestic MCI (naMCI). While both subtypes share common neuropathological features, such as medial temporal lobe atrophy and white matter abnormalities (Pihlajamaki et al., 2009), aMCI is associated with more severe disruptions in brain structural connectivity. The core clinical manifestation of aMCI is episodic memory impairment, typically demonstrated by performance scores 1–1.5 standard deviations below the mean of age- and education-matched peers on relevant tests (Albert et al., 2011; Sun et al., 2018). Crucially, aMCI is considered a high-risk prodromal stage of Alzheimer’s disease (AD), as it often exhibits the characteristic pathological hallmark of amyloid-beta deposition and has a high rate of progression to full-blown AD (Kasper et al., 2020; Yeung et al., 2022; Aerts et al., 2017; Vettore et al., 2021). Therefore, the early identification of aMCI is pivotal for the timely diagnosis and intervention of AD.

The clinical manifestations and conversion risk of aMCI are deeply rooted in the large-scale network organization of the human brain. The functional capabilities of the human brain rely on vast numbers of neurons distributed across distinct brain regions. These regions form extensive structural connections through white matter fiber tracts, such as the corpus callosum and arcuate fasciculus. On this structural foundation, the synchronization of neural activity gives rise to complex functional connections between regions. In recent years, the brain network research paradigm has emerged as a central approach for understanding high-level cognitive functions. This paradigm posits that brain function is largely dependent on its underlying structural framework. Consequently, the integrated analysis of structural and functional connectivity is crucial. Technically, structural networks are typically quantified using Diffusion Tensor Imaging (DTI) to trace the orientation and density of white matter tracts, while functional networks are parsed using resting-state functional MRI (rs-fMRI) to analyze temporal co-activation patterns across brain regions. These two methodological approaches are complementary, and together, they provide a powerful analytical framework for uncovering the neural mechanisms underlying brain disorders such as Mild Cognitive Impairment.

Within this framework, a body of neuroimaging studies has demonstrated widespread multi-network abnormalities in patients with aMCI, with particularly prominent involvement of the default mode network (DMN), salience network, and dorsal attention network (Wu et al., 2023; Song et al., 2021; Quattrini et al., 2021; Xue et al., 2021; Zhou et al., 2022). Specifically, the DMN exhibits complex alterations in resting-state functional connectivity (Eyler et al., 2019), characterized not only by reduced within-network connectivity but also by hyperconnectivity between the DMN and certain other brain regions (Wang et al., 2019; Lee et al., 2023). Beyond these functional disruptions, studies have further identified impaired white matter structural networks and reduced neural activity levels in aMCI, both of which correlate significantly with the degree of cognitive impairment (Shao et al., 2021; Chen et al., 2022). In this context, the integration of multi-modal imaging techniques—such as fMRI and DTI—to consolidate features across different network levels provides a powerful means to elucidate the mechanisms underlying cognitive deficits from MCI to Alzheimer’s disease (Xu et al., 2022). Consequently, in-depth analysis of brain network architecture serves not only as a foundation for understanding cerebral function but also as a critical avenue for clarifying the pathological mechanisms of cognitive disorders such as aMCI (Chen et al., 2017).

To achieve this in-depth analysis, robust quantitative tools are required. In this context, imaging connectomics and its core analytical method—graph theory—play a crucial role. Imaging connectomics has opened new avenues for exploring human brain organization and function by assessing structural and functional connectivity patterns between regions, with graph theory analysis emerging as a core tool for characterizing the complex topological properties of brain networks (Bullmore and Sporns, 2009). This approach models the brain as a complex network system consisting of nodes and edges (which can be binarized or weighted) (Bertolero et al., 2018), enabling quantitative analysis of network topology from both global and nodal perspectives. Key global metrics include small-world, rich-club, network efficiency (global and local efficiency), among others. Extensive studies have demonstrated that these graph-theoretic measures effectively capture network abnormalities in individuals with MCI. Compared to healthy controls, MCI patients show significant alterations in parameters such as the clustering coefficient, degree centrality, and global and local efficiency, which are closely associated with cognitive decline and impairments in daily living activities (Luo et al., 2021). In structural networks, Zdanovskis et al. (2021) reported that white matter networks in MCI patients exhibited significant differences in characteristic path length, small-world properties, global efficiency, and rich-club organization compared to cognitively normal individuals and dementia patients, suggesting these metrics as potential biomarkers for cognitive impairment progression. Similarly, other studies have confirmed disrupted organization in morphological networks in MCI, manifesting as increased lambda and decreased global efficiency (Chen et al., 2024). In functional networks, graph theory analyses have also revealed distinct alterations in MCI. For instance, Zhang et al. (2020) and Xiong et al. (2021) observed abnormal nodal centrality in functional networks at the MCI stage: degree centrality (DC) was decreased in the left inferior temporal gyrus but increased in the right fusiform gyrus. Moreover, global metrics and modular organization in functional networks were also significantly altered. These characteristics provide valuable imaging evidence for assisting in the early diagnosis of MCI.

Building upon the potential of graph theory to reveal network abnormalities and the inconsistent findings in the existing literature, this study aims to systematically investigate topological disruptions in aMCI patients. Specifically, based on PANDA and DPABI software, this study constructed white matter structural and resting-state functional networks for two groups of participants. Graph theory was applied to analyze global topological properties, including small-world, Rich-club, network efficiency, assortativity, hierarchy, and synchronization. Combined with the “disconnection hypothesis”(Palesi et al., 2016) and “compensatory theory”(Behfar et al., 2020), this study systematically verified the pattern differences in bimodal network topological disruptions in aMCI patients and revealed significant correlations between multimodal topological indices and clinical cognitive scores, thereby providing multimodal network evidence for elucidating the pathological mechanisms of aMCI.

## 2 Materials and methods

Previous studies have shown that in imaging research, larger sample sizes lead to more reliable results (Chen et al., 2019). However, in actual experiments, the high cost of magnetic resonance sequence scanning and the difficulty in subject cooperation have resulted in relatively small sample sizes in most current neuroimaging studies, typically controlled at around 20 cases. Based on this, this trial collected 45 healthy elderly subjects from the First Affiliated Hospital of Heilongjiang University of Chinese Medicine as the control group (HC group), and 45 amnestic MCI patients as the experimental group (aMCI group). However, in the subsequent data processing and calculation, data from 3 subjects in the HC group were excluded. All subjects were aged 55–75 years, right-handed, with a history of good physical health, no mental diseases, and no family genetic history; they had no dietary history of excessive consumption of foods affecting central nervous excitability such as coffee and tea. There was no statistically significant difference in general data between the groups.

### 2.1 Inclusion criteria

#### 2.1.1 Inclusion criteria for the aMCI

➀ Aged between 55 and 75 years old; ➁ Selection of individuals without bad habits, such as non-smoking and non-alcoholism; ➂ Self-reported or reported by others to have mild memory problems; ➃ The scoring criteria of the Mini-Mental State Examination (MMSE) scale: for the illiterate group, the score is ≥17 points; for the primary school education group, the score is ≥20 points; for the group with junior high school education or above, the score is ≥24 points. In the tests of memory or other cognitive domains, the performance is lower than that of peers but higher than 1.5 standard deviations below the standard score, and the score of the Clinical Dementia Rating (CDR) scale is 0.5 points; ➄ The total score of the Montreal Cognitive Assessment (MoCA) scale is ≤26 points, and other diseases that can cause cognitive impairment, such as brain trauma, stroke, Parkinson’s disease, and hypothyroidism, are excluded; ➅ The results of cranial MRI examination are normal, and the skin at the acupuncture site is intact without scars.

#### 2.1.2 Inclusion criteria for the HC

➀ No complaints of cognitive impairment; ➁ Normal neurological examination;➂ MMSE score >26, and multidimensional neuropsychological scores within the normal range matched for age or educational level; ➃ Ability to cooperate with fMRI scanning; ➄ No history of depression, anxiety, or other mental symptoms.

### 2.2 Exclusion criteria

(1)    Obvious dementia;
(2)    Having impairments such as visual, auditory, or aphasic disorders, or other severe cognitive impairments, such as Alzheimer’s disease, Parkinson’s disease, etc.;
(3)    Suffering from major mental illnesses or severe visceral dysfunctional diseases of the heart, liver, kidneys, etc.;
(4)    Long-term use of drugs that affect cognitive abilities, such as benzodiazepines, anticholinergic drugs, etc.; or those who have taken sedative drugs within the last month before the trial; or having diseases that may affect cognitive performance, such as epilepsy, brain tumors, severe head trauma, etc.;
(5)    Drug abuse or alcoholism;
(6)    Having a pacemaker or metal fragments in the body that affect MRI scanning;
(7)    Having psychological diseases such as claustrophobia and being unable to undergo MRI scanning;
(8)    Congenital cranial malformation;
(9)    Color blindness, etc.

### 2.3 Withdrawal criteria

(1)    During scanning, head movement amplitude exceeds the allowable error range. Specifically, in the head motion correction preprocessing stage, if the displacement of any frame of images in the HC group exceeds 2.0 mm or the rotation angle is greater than 2°, the subject’s data will be excluded.
(2)    During the trial, the subject fails to complete all experimental procedures as required by the protocol, such as withdrawing without authorization or refusing to cooperate with specific examinations/tests.
(3)    Sudden equipment failure during data acquisition leads to missing, corrupted, or severely abnormal data that cannot be repaired by technical means.
(4)    Incomplete image scanning coverage due to incorrect machine parameter settings during the trial, resulting in exclusion of the image data.

### 2.4 Experimental equipment

The experiment used a Philips Ingenia 3.0T fully digital MRI scanner with a gradient field strength of 40 mT/m, a 16-channel parallel head coil (SENSE-NV-160), and an 80 MHz high-frequency analog-to-digital converter for each channel, eliminating the need for analog filtering through direct digital sampling. The gradient switching rate was 200 mT/m/ms.

### 2.5 Preparation before the experiment

Most of the subjects have not experienced acupuncture and cranial magnetic resonance examination, and their psychological state may affect the accuracy of the experiment. Before the experiment, the process was informed to relieve their anxiety. During the experiment: ➀ The subjects were required to remove metal objects and items that could interfere with the magnetic field and change into the experimental clothing; ➁ Earplugs and eye masks were worn after entering the magnetic field; ➂ The subjects lay supine on the scanning table, placed their heads in the coil, and remained still. If the subjects wanted to terminate the experiment, they could wave to indicate.

### 2.6 Imaging data acquisition

Before performing fMRI (FFE single-shot EPI) and DTI sequence (SE single-shot EPI) scans, all subjects underwent routine sequence scans (T1WI_TFE_ref SENSE, T2WI_FLAIR SENSE) and 3D-T1WI sequence scans (T1WI-3D-TFE-ref). The purpose was to exclude brain diseases and facilitate spatial registration of fMRI and DTI images. The specific parameters for the different sequences are provided in Table 1.

**TABLE 1:** Scanning parameters of the experimental sequence.

Parameters	T1WI_TFE_ref SENSE	T2WI_FLAIR SENSE	T1WI-3D-TFE-ref	SE single-shot EPI	FFE single-shot EPI
TR	1500 ms	2224 ms	7.4 ms	3516 ms	2000 ms
TE	24 ms	80 ms	3.4 ms	84 ms	30 ms
Matrix	320 × 256	368 × 242	256 × 256	96 × 96	64 × 64
FOV	240 × 240 mm	240 × 240 mm	256 × 256 × 288 mm	224 × 224 × 138 mm	220 × 220 × 143 mm
Slice num	24	24	188	60	36
SG	1 mm	1 mm	0 mm	0 mm	1 mm
ST	6 mm	6 mm	1 mm	2 mm	3 mm
FA	–	90°	8°	–	90°
Number of excitations	1	1	1	–	–
Scan time	2 min 19.7 s	1 min 24.5 s	4 min 52 s	5 min 13 s	6 min 6 s

TR, repetition time; TE, echo time; FOV, field of view; SG, slice gap; ST, slice thickness; FA, flip angle.

### 2.7 Imaging data preprocessing

#### 2.7.1 DTI data

For the HC and aMCI groups, DTI sequence scans were acquired. DTI image data were preprocessed and DTI indices were calculated using the VBA method in the PANDA software (based on the Linux system), and whole-brain white matter structural networks were constructed via deterministic fiber tracking. PANDA is a toolbox for analyzing and calculating brain DTI images, integrating the functions of FSL, Diffusion Toolkit, and MRIcron software. The specific steps were as follows: ➀ Convert the original DTI images of all subjects from DICOM format to NIFIT format; ➁ Remove non-brain tissues such as scalp and skull via professional algorithms to construct brain mask images; ➂ Perform eddy current correction and head motion correction on images to eliminate distortion caused by motion and eddy currents; ➃ Calculate DTI indices for each subject; ➄ Non-linearly register and normalize subject images to the Montreal Neurological Institute (MNI) standard space; ➅ Perform image smoothing and standardization.

#### 2.7.2 FMRI data

First, MRIconvert software was used for data conversion, transforming the original DICOM format data into NIFTI format. Subsequently, preprocessing of the converted NIFTI data was performed using the Data Processing Assistant for Resting-State fMRI (DPARSFA) on the MATLAB platform, with specific steps as follows: ➀ Discard images from the first 10 time points. ➁ Perform slice timing correction between layers. ➂ Conduct realignment for head motion correction between points. ➃ Spatial registration. ➄ Segment gray matter, white matter, and cerebrospinal fluid in 3D structural images. ➅ Spatial normalization. ➆ Covariate regression. ➇ Smoothing. ➈ Detrending. ➀0 Filtering.

### 2.8 Brain network construction

**White Matter Structural Network:** The PANDA software package was used to construct the brain white matter structural network based on the preprocessed results. The two basic elements of the network are nodes (N) and edges (E). The specific steps for constructing the whole-brain structural network in this study were as follows: ➀ Node Definition: The Automated Anatomical Labeling (AAL) template was used to partition the cerebrum (excluding the cerebellum) into 90 regions, which served as network nodes. The calculated diffusion tensor indices were registered to the standard FMRIB58_FA template provided by FSL. ➁ Edge Definition: The deterministic fiber tracking algorithm in PANDA was employed for whole-brain white matter fiber tractography, with tracking terminated when the deflection angle exceeded 45° or the fractional anisotropy (FA) value of a voxel was <0.2. The mean FA value of the fiber tracts connecting each pair of nodes was extracted to form the edge matrix (FA matrix).

Based on the Matlab2017b platform under the Windows operating system, the Gretna (Graph Theoretical Network Analysis) software package was used for graph theory-based analysis of white matter structural networks (Wang et al., 2015). To ensure the validity of structural network connections and avoid spurious connections in the FA matrix, within a specific range of sparsity (Sparsity, S), an undirected binary matrix was constructed, and the absolute values of connection edges were used to calculate the topological properties of the structural network in this study. The final determined sparsity analysis range is 0.21–0.41 (with an interval of 0.01): the lower limit of 0.21 is calculated by the criterion that the average node degree > 2 × ln (N) (where *N* = 90 represents the number of node brain regions), ensuring network connectivity; the upper limit of 0.41 is set with reference to the conventional sparsity range in the field and pre-experiments. This threshold range maximally ensures fully connected structural networks with minimal spurious edges, and guarantees that the Sigma >1 and the normalized rich-club coefficient >1.

**Functional Network:** This study reconstructed the functional network of subjects using the Gretna package (Wang et al., 2015). The adjacent subcortical regions were divided into 116 nodes according to the AAL template and registered. After extracting the average time series of each node, Pearson correlation coefficients were calculated to construct a matrix, which was transformed into a Z matrix via Fisher’s transformation. By setting a sparsity threshold, the matrix was converted into an undirected binary matrix, incorporating both positive and negative functional connections. The threshold range exhibiting small-world properties (high clustering coefficient and short path length) was selected to exclude spurious connections. This approach abstracts the brain network as nodes and edges using graph theory, and combines the characteristics of positive/negative connections with small-world properties to effectively analyze the topological structure of functional interactions among brain regions. This method utilizes graph theory to abstract and simplify brain networks into nodes and edges, enabling effective analysis of the topological structure of functional interactions between brain regions.

For structural networks, we employed the 90-node AAL atlas, focusing on major cortical and subcortical regions to ensure the reliability of DTI-based tractography. For functional networks, the complete 116-node AAL atlas was used to capture the full repertoire of whole-brain functional connectivity patterns, including the cerebellum. This differential atlas selection represents an optimization tailored to the technical specifics and research requirements of each modality.

### 2.9 Topological property metrics of brain networks

(1)    Small-world. Small-world networks are a class of special network models whose properties lie between regular networks and random networks (Liao et al., 2017). The small-world property metrics calculated in this study include clustering coefficient (C_*p*_), Characteristic Path Length (L_*p*_), Gamma (γ), Lambda (λ), and Sigma (σ). The conditions for small-world properties are: Gamma > > 1; Lambda ≈ 1; Sigma >1.
(2)    Rich-club (Griffa and Van den Heuvel, 2018). For the Rich-club, the primary evaluation is the Rich-club coefficient, which is uniformly standardized. When the results show that the φnorm >1 within a range of node degrees (*K*), it is considered that the brain has Rich-club organization. In this study, the top 20 brain regions with the highest nodal metrics were defined as rich-club nodes, and the remainder were designated as peripheral (non-rich-club) nodes (Daianu et al., 2013). In the WM structural network, edges are defined by network connection density, which is the proportion of the number of connected edges to the total possible edges. In the functional network, edges are defined by connection strength, a continuous weighted value (Pearson correlation coefficient) reflecting the intensity of activity synchrony between brain regions. Rich-club has three types of edges: connections between high-degree nodes are rich-club connections; connections between high-degree and low-degree nodes are feeder connections; connections between low-degree nodes are local connections. The brain’s Rich-club network and non-Rich-club network were constructed accordingly.
(3)    Network Efficiency: Efficiency is one of the most commonly used and core indices in brain network research, widely applied to structural and functional network studies. Global efficiency (E_*g*_) is the average of the reciprocals of the shortest paths between any pair of nodes, reflecting the overall information transmission efficiency of the network. Compared with the L_*p*_, E_*g*_ avoids the case of infinite paths caused by disconnected nodes in the network. Local efficiency (E_*loc*_) is the mean of the local efficiencies of all nodes, where the local efficiency of a node is defined as the average of the reciprocals of the shortest paths in the subnetwork formed by its neighbors. Thus, E_*loc*_ reflects the information transmission efficiency of each local region in the network.
(4)    Assortativity: It is used to examine whether nodes with similar degrees tend to connect with each other, serving as an indicator of network resilience (Javaheripour et al., 2023). If nodes with high degrees generally tend to connect with other high-degree nodes, the network is said to have positive degree correlation or be assortative. Conversely, if high-degree nodes generally tend to connect with low-degree nodes, the network is said to have negative degree correlation or be disassortative (Peel et al., 2018). The assortativity coefficient (*r*) is a Pearson correlation coefficient based on “degree” to measure the relationship between connected node pairs. A positive *r* indicates a synergistic relationship between nodes with the same degree, while a negative *r* indicates connections between nodes with different degrees. The *rzscore*, a standardized modification of the assortativity coefficient *r*, is calculated as (actual network *r* - average *r* of random networks) divided by the standard deviation of *r* across random networks. Its function is to eliminate baseline interference from random networks and quantify the deviation of the actual network’s assortativity or disassortativity from that of random networks—specifically, a larger *rzscore* indicates a more pronounced assortative or disassortative property of the network relative to random networks.
(5)    Hierarchy: Hierarchy is a principle followed by brain networks and a crucial metric describing the directionality of information processing, nested relationships among functional modules, and structure-function gradients (Yin et al., 2024). It is not a single value but reveals the ordered hierarchical structure of the network from “lower-level” sensory processing to “higher-level” cognitive integration through a series of calculations (Murphy et al., 2018). In hierarchically structured networks, nodes with high degrees typically have lower clustering coefficients, while those with low degrees have higher clustering coefficients, exhibiting a scaling relationship between node degree and clustering coefficient. Therefore, the hierarchical index *b* can be computed by fitting the linear relationship between the logarithms of node clustering coefficients and node degrees to quantify the network’s hierarchical structure (Ravasz and Barabasi, 2003). The *b* reflects the presence of hierarchical organization: A higher value of *b* indicates a clearer hierarchical structure in the brain network; conversely, a lower *b* value implies more random and scattered connections between network nodes. This can be understood as follows: When a decrease in *b* is accompanied by significant progressive cognitive decline—particularly in functions related to network integration such as executive function, information processing speed, and episodic memory—such reduced hierarchy may reflect early pathological changes in the network. If *b* decreases but cognitive function remains intact, it is inferred that the brain network achieves effective compensation through other mechanisms (e.g., enhanced local connections). The *b*zscore is a standardized modification of *b*, with calculation methods and functions consistent with the assortativity coefficient. Specifically: *b*zscore >0 indicates that the hierarchy of the actual network is significantly higher than that of random networks, with clearer hierarchical division of labor; *b*zscore ≈ 0 means the hierarchy of the actual network is close to that of random networks; *b*zscore <0 denotes that the hierarchy of the actual network is significantly lower than that of random networks, with more disorganized hierarchical division of labor.
(6)    Synchronization: In the WM structural network, s denotes the global structural connection strength or average structural connectivity, reflecting the overall density or total intensity of connections. A higher s value indicates a more densely connected and robust WM structural network, while a lower s value implies relatively sparser or weaker overall structural connections. It serves as a critical potential structural basis and supporter for functional synchronization (Choi and Mihalas, 2019). In functional networks, s represents global functional synchrony, a specific quantitative index of brain network synchronization, designed to measure the temporal consistency of activities across all brain regions. A high s value signifies high global brain activity synchrony (i.e., strong overall integration), whereas a low s value indicates weak synchrony (i.e., weak integration and strong differentiation).

### 2.10 Statistical analysis

Clinical and imaging data of subjects, including general demographics (age, gender, years of education) and neuropsychological scale scores, were analyzed using SPSS 23.0 statistical software, with data presented as mean ± standard deviation (SD). Chi-square test, two-sample *t*-test, and Mann-Whitney *U* test were used for intergroup comparisons. A *p*-value <0.05 was considered statistically significant. Results were visualized using Prism 10.1.2 and OriginPro2024 software.

## 3 Results

### 3.1 General data

There were no significant statistical differences between the aMCI and HC groups in demographics (age, gender, years of education). However, there were significant statistical differences in neuropsychological scores (MMSE, MoCA) (*P* < 0.05). See Table 2 for details.

**TABLE 2:** Comparison of demographic and neuropsychological scores between the aMCI and HC groups.

Category	HC (*n = 42*)	aMCI (*n = 45*)	*P-*values
**Demography**			
Age	62.95 ± 5.55	65.04 ± 8.37	0.176
Sex (male: female)	18:24	17:28	0.790
Education level (years)	11.71 ± 3.20	10.36 ± 4.47	0.109
**Neuropsychological score**			
MMSE (points)	28.26 ± 1.06	25.20 ± 1.21	0.000
MoCA (points)	27.36 ± 1.21	22.42 ± 4.40	0.000

*P* < 0.05 indicates statistical significance. Categorical variables (gender) were analyzed using the χ*^2^* test; normally distributed data were analyzed using an independent sample *t*-test (mean ± standard deviation); non-normally distributed data were analyzed using the Mann-Whitney *U* test [median (interquartile range)]. HC, healthy controls; aMCI, amnestic mild cognitive impairment; MMSE, Mini-Mental State Examination; MoCA, Montreal Cognitive Assessment.

### 3.2 Topological property of brain Networks

#### 3.2.1 Small-world

(1) WM structural network

**FIGURE 1:**
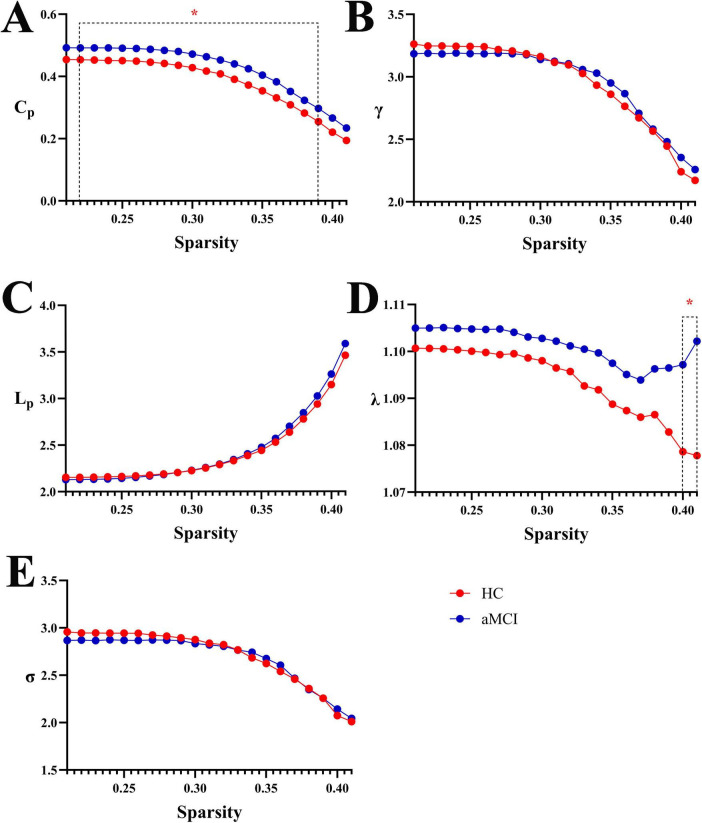
Trend graphs of small-world property indices in WM structural networks of HC and aMCI groups across sparsity thresholds of 0.21∼0.41. (A) Line graph comparing C_p_ values between groups across sparsity; (B) line graph comparing Gamma (γ) values between groups across sparsity; (C) line graph comparing L_p_ values between groups across sparsity; (D) line graph comparing Lambda (λ) values between groups across sparsity; (E) line graph comparing Sigma (σ) values between groups across sparsity. C_p_: clustering coefficient; Gamma: normalized clustering coefficient; Lambda: normalized characteristic path length; L_p_: characteristic path length; Sigma: small-world coefficient. *Indicating statistical significance, *P* < 0.05 (independent-samples *t*-test).

By analyzing the trend graphs of small-world property indices (C_*p*_, γ, λ, L_*p*_, σ) in HC and aMCI groups across sparsity thresholds of 0.21 to 0.41, both groups exhibited small-world properties before and after acupuncture. Specifically, L_*p*_ significantly increased with rising sparsity, while γ and σ significantly decreased. Notably, HC and aMCI groups showed significant differences in C_*p*_ across the entire 0.21∼0.39 sparsity range (*P* < 0.05), and in λ at sparsity thresholds of 0.40 (*P* < 0.05). See Figure 1.

**FIGURE 2:**
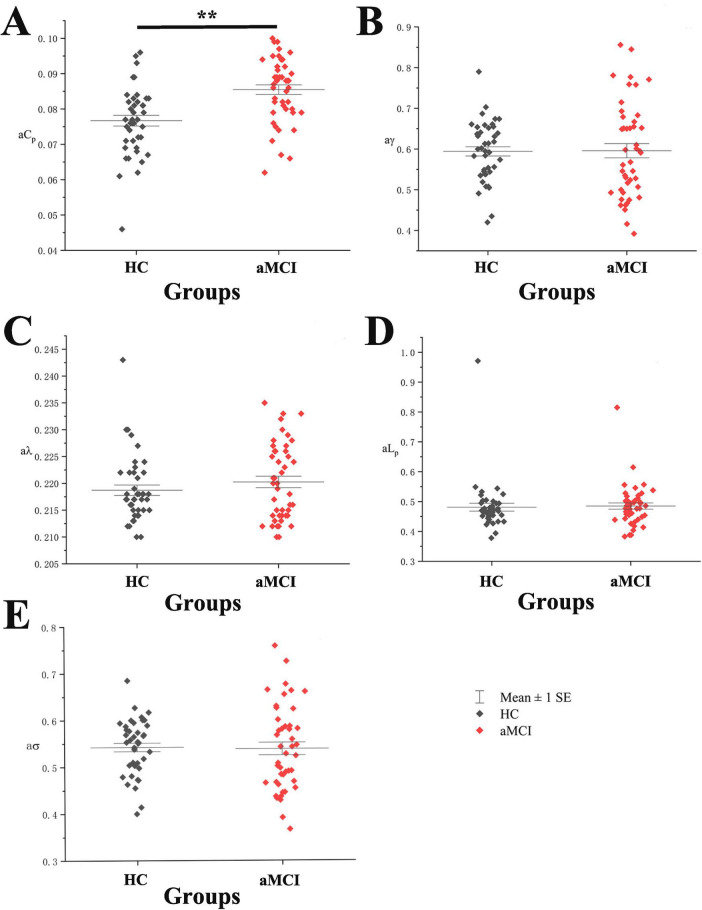
Boxplot with scatter points comparing total AUC of WM structural network small-world property indices. (A) It shows a scatter-box plot comparing the AUC of the WM structural network’s C_*p*_ between the two groups of subjects. (B) It shows a scatter-box plot comparing the AUC of the WM structural network’s g between the two groups of subjects. (C) It shows a scatter-box plot comparing the AUC of the WM structural network’s / between the two groups of subjects. (D) It shows a scatter-box plot comparing the AUC of the WM structural network’s L_*p*_ between the two groups of subjects. (E) It shows a scatter-box plot comparing the AUC of the WM structural network’s s between the two groups of subjects. **Indicating statistical significance, *P* < 0.01 (independent-samples *t*-test). Mean ± SE: Mean ± Standard Error.

Statistical analysis of the total area under the curve (AUC) for small-world property indices (aC_*p*_, aγ, aλ, aL_*p*_, aσ) in HC and aMCI groups showed a significant difference in aC_*p*_ between aMCI and HC groups (*P* < 0.05), with no significant differences in other indices. See Figure 2.

(2) Functional network

**FIGURE 3:**
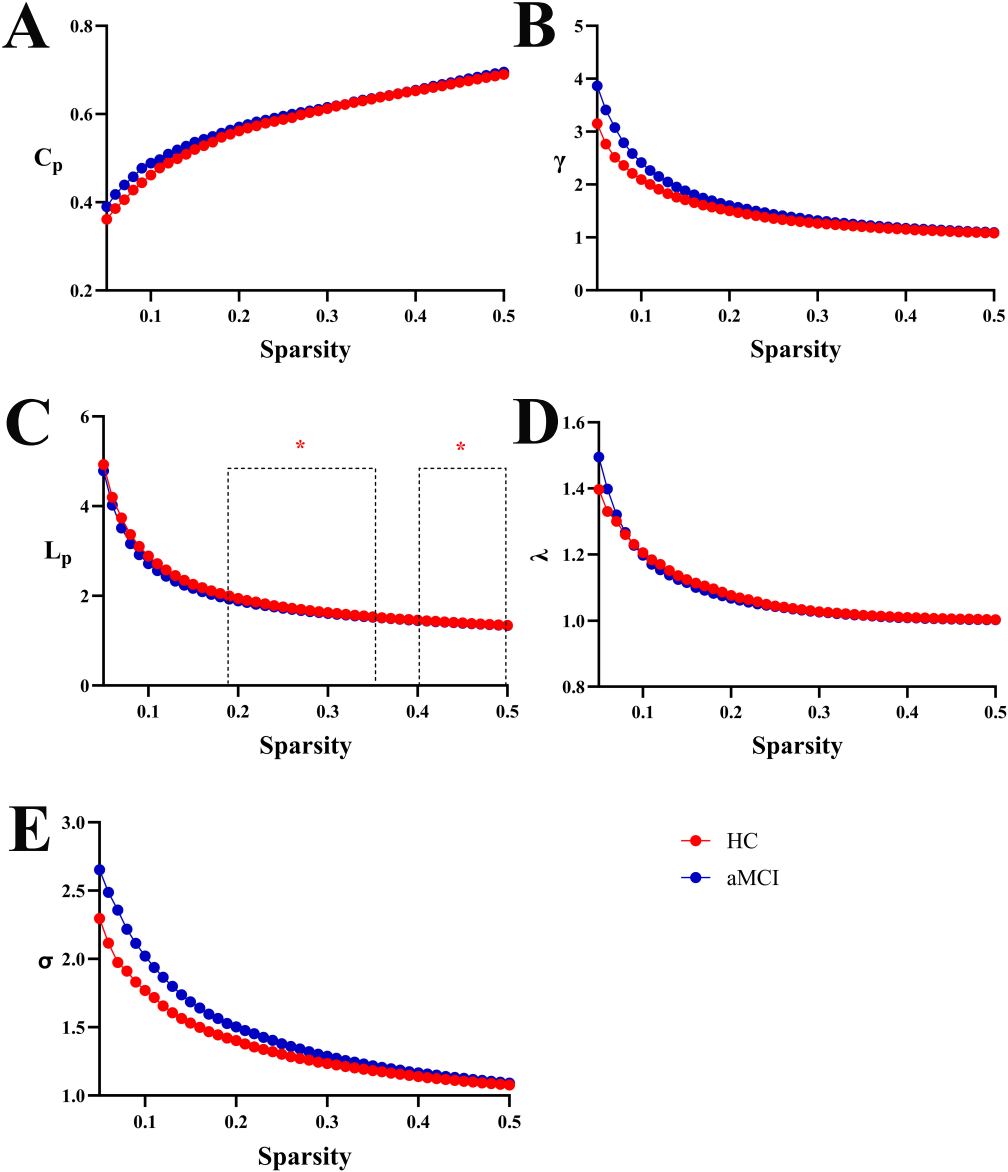
Trend graphs of small-world property indices in functional networks of HC and aMCI groups across sparsity thresholds of 0.05–0.5. (A) Line graph comparing C_p_ values between groups across sparsity; (B) line graph comparing Gamma (γ) values between groups across sparsity; (C) line graph comparing L_p_ values between groups across sparsity; (D) line graph comparing Lambda (λ) values between groups across sparsity; (E) line graph comparing Sigma (σ) values between groups across sparsity. *Indicating statistical significance, *P* < 0.05 (independent-samples *t*-test).

By analyzing the trend graphs of small-world property indices (C_*p*_, γ, λ, L_*p*_, σ) in HC and aMCI groups across sparsity thresholds of 0.05 to 0.5, both groups exhibited small-world properties. γ, λ, L_*p*_, and σ showed decreasing trends with increasing sparsity. Notably, significant group differences were observed in: L_*p*_ across sparsity 0.18∼0.35 and 0.4∼0.5 (*P* < 0.05). No significant differences were found in C_*p*_, γ, λ and σ across sparsity. See Figure 3.

**FIGURE 4:**
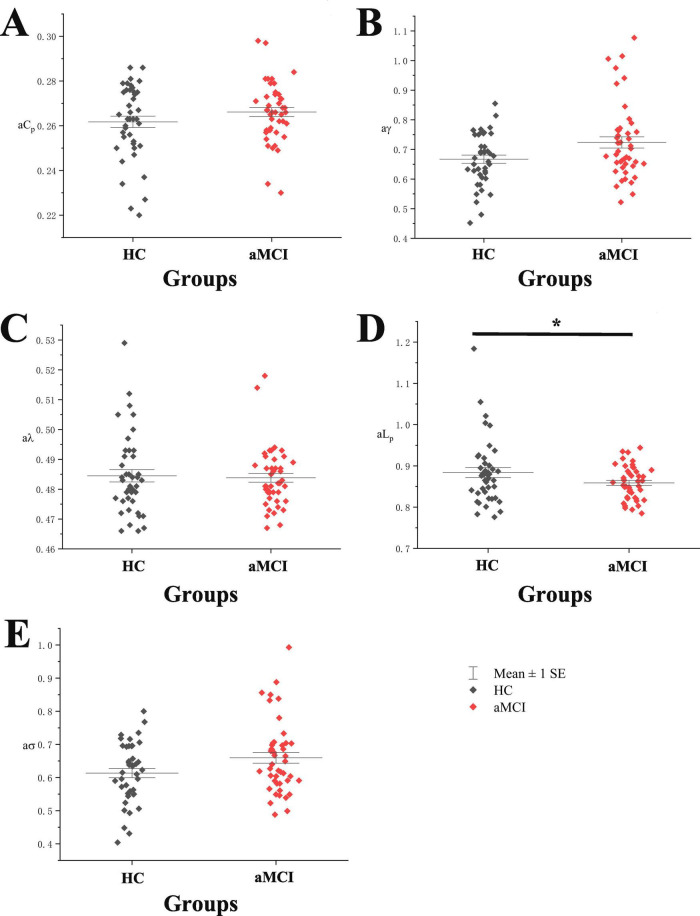
Boxplot with scatter points comparing total AUC of functional network small-world property indices. (A) It shows a scatter-box plot comparing the AUC of the functional network’s C_*p*_ between the two groups of subjects. (B) It shows a scatter-box plot comparing the AUC of the functional network’s g between the two groups of subjects. (C) It shows a scatter-box plot comparing the AUC of the functional network’s/between the two groups of subjects. (D) It shows a scatter-box plot comparing the AUC of the functional network’s L_*p*_ between the two groups of subjects. (E) It shows a scatter-box plot comparing the AUC of the functional network’s s between the two groups of subjects. *Indicating statistical significance, *P* < 0.05 (independent-samples *t*-test). Mean ± SE: Mean ± Standard Error.

Statistical analysis of the total AUC for small-world property indices (aC_*p*_, aγ, aλ, aL_*p*_, aσ) in HC and aMCI groups revealed significant differences in aL_*p*_ between aMCI and HC groups (*P* < 0.05), with no significant differences in other indices. See Figure 4.

#### 3.2.2 Rich-club

(1) WM structural network

**FIGURE 5:**
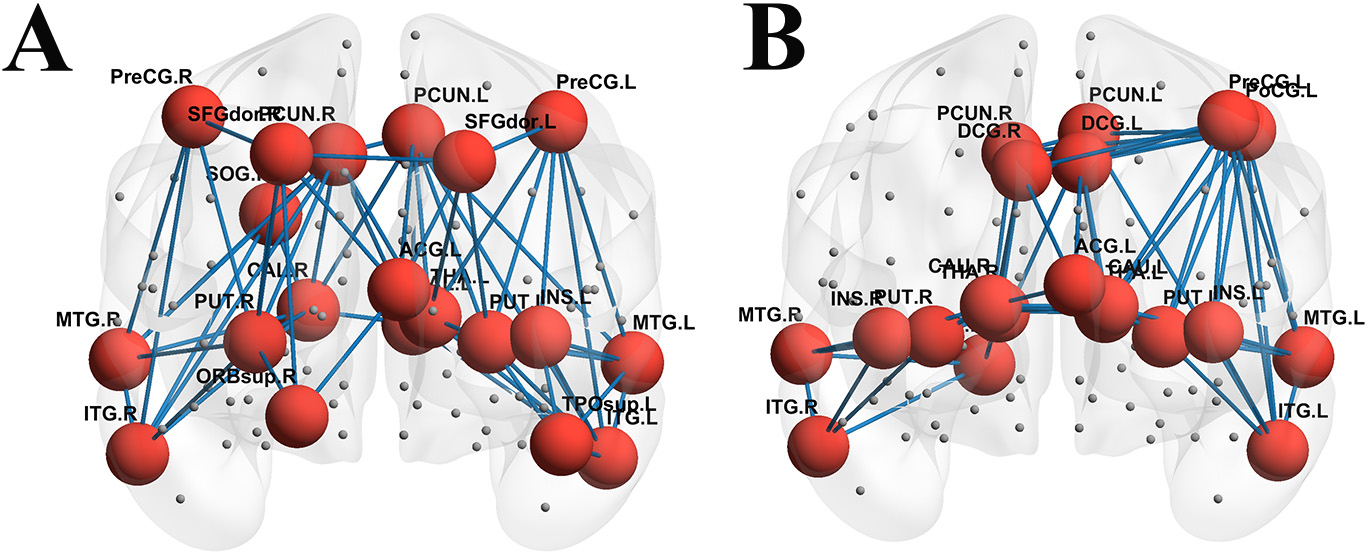
Structural network Rich-club organization. **(A)** Rich-club connections in the HC group; **(B)** Rich-club connections in the aMCI group; red spheres represent Rich-club nodes; gray spheres represent edge nodes (non-Rich-club nodes).

Based on the WM structure matrix, the top 20 nodes with the highest degree values were identified for each of the two groups. It was found that a redistribution of nodes with high degree values occurred in the structural networks between the two groups, and the details are presented in Table 3. Additionally, compared with the HC group, the aMCI group showed differences in Rich-club connections, as illustrated in Figure 5.

**TABLE 3:** Distribution of rich-club nodes in structural and functional networks for the aMCI and HC groups.

Rank	WM network (AAL90)		Function network (AAL116)		Degree (K)
	HC group	aMCI group	HC group	aMCI group	
1	74. Putamen_R	33. Cingulum_Mid_L	67. Precuneus_L	67. Precuneus_L	*K* ≥ 1
2	68. Precuneus_R	73. Putamen_L	99. cerebelum_6_L	68. Precuneus_R	*K* ≥ 2
3	73. Putamen_L	68. Precuneus_R	68. Precuneus_R	48. Lingual_R	*K* ≥ 3
4	67. Precuneus_L	67. Precuneus_L	100. cerebelum_6_R	70. Paracentral_Lobule_R	*K* ≥ 4
5	89. Temporal_Inf_L	30. Insula_R	82. Temporal_Sup_R	56. Fusiform_R	*K* ≥ 5
6	90. Temporal_Inf_R	34. Cingulum_Mid_R	48. Lingual_R	55. Fusiform_L	*K* ≥ 6
7	44. Calcarine_R	90. Temporal_Inf_R	91. Cerebelum_Crus1_L	99. Cerebelum_6_L	*K* ≥ 7
8	86. Temporal_Mid_R	74. Putamen_R	20. Supp_Motor_Area_R	51. Occipital_Mid_L	*K* ≥ 8
9	2. Precentral_R	71. Caudate_L	85. Temporal_Mid_L	90. Temporal_Inf_R	*K* ≥ 9
10	4. Frontal_Sup_R	89. Temporal_Inf_L	51. Occipital_Mid_L	47. Lingual_L	*K* ≥ 10
11	50. Occipital_Sup_R	29. Insula_L	86. Temporal_Mid_R	100. Cerebelum_6_R	*K* ≥ 11
12	83. Temporal_Pole_Sup_L	31. Cingulum_Ant_L	69. Paracentral_Lobule_L	69. Paracentral_Lobule_L	*K* ≥ 12
13	77. Thalamus_L	85. Temporal_Mid_L	56. Fusiform_R	86. Temporal_Mid_R	*K* ≥ 13
14	85. Temporal_Mid_L	72. Caudate_R	52. Occipital_Mid_R	20. Supp_Motor_Area_R	*K* ≥ 14
15	3. Frontal_Sup_L	57. Postcentral_L	55. Fusiform_L	43. Calcarine_L	*K* ≥ 15
16	43. Calcarine_L	48. Lingual_R	70. Paracentral_Lobule_R	2. Precentral_R	K ≥ 16
17	31. Cingulum_Ant_L	1. Precentral_L	81. Temporal_Sup_L	91. Cerebelum_Crus1_L	*K* ≥ 17
18	29. Insula_L	86. Temporal_Mid_R	46. Cuneus_R	16. Frontal_Inf_Orb_R	K ≥ 18
19	1. Precentral_L	77. Thalamus_L	90. Temporal_Inf_R	4. Frontal_Sup_R	*K* ≥ 19
20	6. Frontal_Sup_Orb_R	78. Thalamus_R	47. Lingual_L	3. Frontal_Sup_L	*K* ≥ 20

**FIGURE 6:**
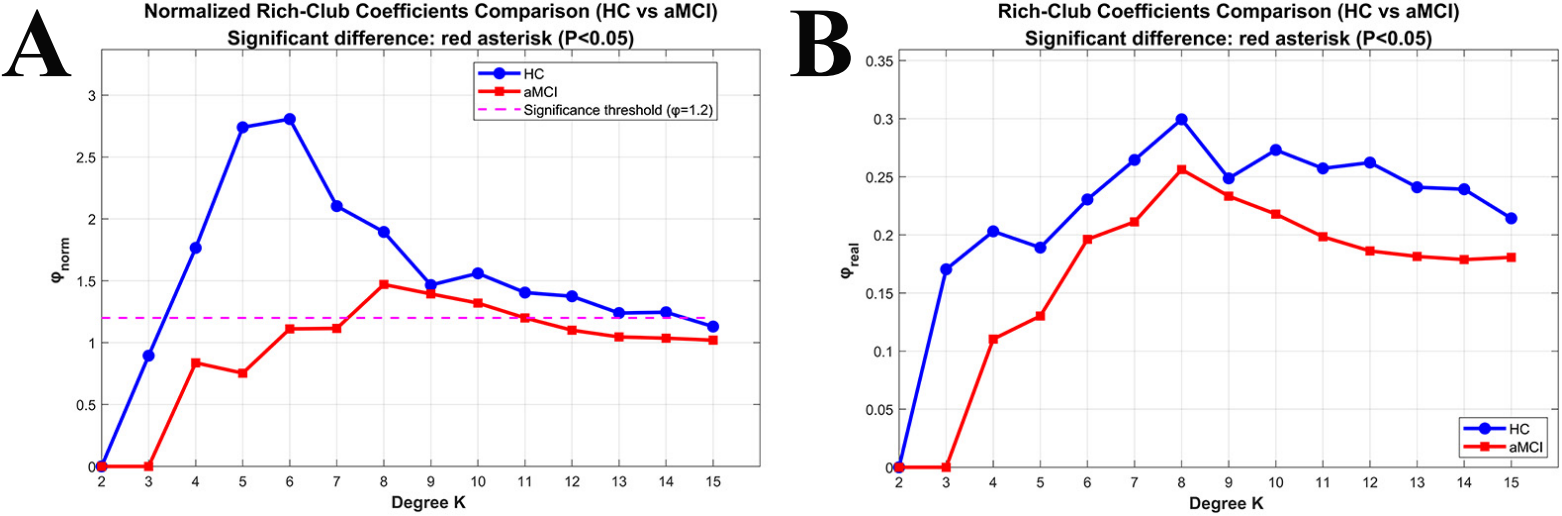
Relationships between Rich-club coefficients (φnorm and φreal) and degree (K) in WM networks. **(A)** Changes in normalized Rich-club coefficients (φnorm) with K between the two groups; **(B)** changes in real Rich-club coefficients (φreal) with K between the two groups.

For *K* = 3 to15, the φnorm of the structural network in the HC group was >1; whereas for the aMCI group, the φnorm was > >1 only when *K* = 6 to15. Moreover, the coefficient of the aMCI group was lower than that of the HC group. Both φnorm and φreal showed no significant difference between the two groups, As shown in Figure 6.

**(2) Functional network**

**FIGURE 7:**
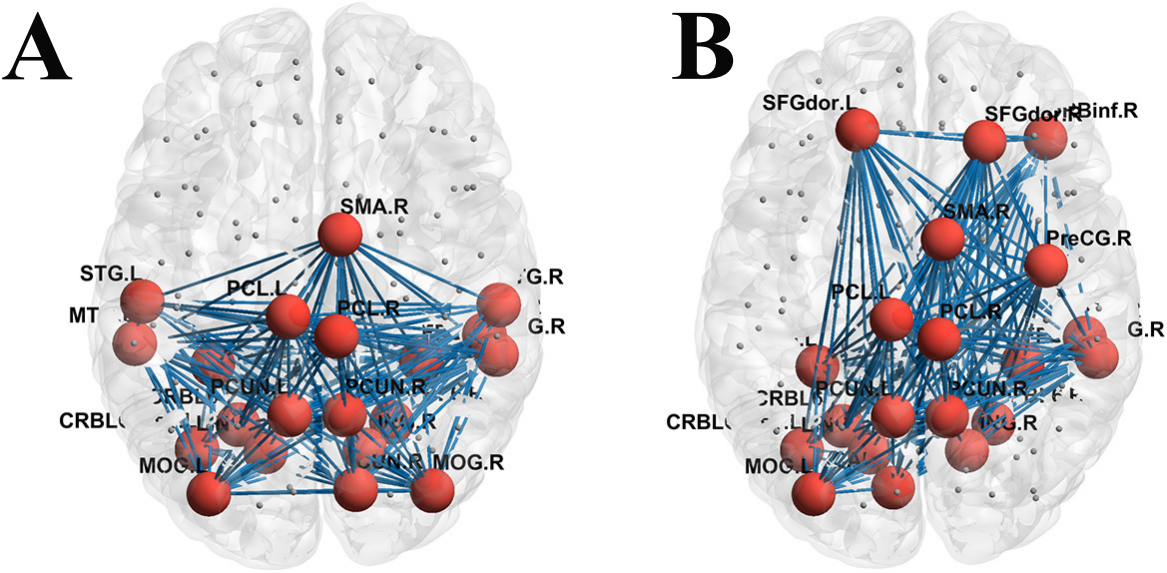
Functional network Rich-club organization. **(A)** Rich-club connections in the HC group; **(B)** Rich-club connections in the aMCI group; red spheres represent Rich-club nodes; gray spheres represent edge nodes (non-Rich-club nodes).

The top 20 nodes with the highest degree values in both groups were derived from the functional matrices, respectively. It was found that the degree-value nodes in the functional networks of the two groups also showed redistribution, yet the number of brain regions with changes was fewer than that in the white matter structural network (see Table 3). Additionally, significant differences in Rich-club connections were observed between the HC group and the aMCI group as shown in Figure 7.

**FIGURE 8:**
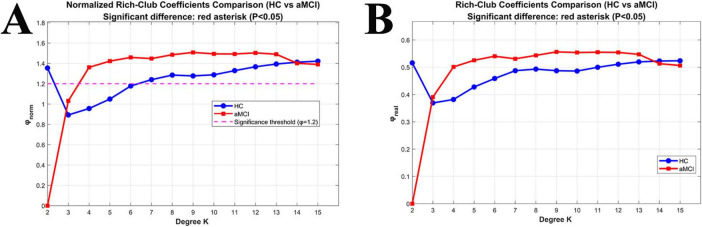
Relationships between Rich-club coefficients and degree (K) in functional networks. **(A)** Changes in normalized Rich-club coefficients (φnorm) with K between the two groups; **(B)** changes in real Rich-club coefficients (φreal) with K between the two groups.

For *K* = 2 to 15, the φnorm of the functional network in the HC group was >1; whereas in the aMCI group, the φnorm of the functional network was >1 when *K* = 3 to 15. Both φnorm and φreal showed no significant difference between the two groups, as shown in Figure 8.

#### 3.2.3 Network efficiency

**(1) WM structural network**

**FIGURE 9:**
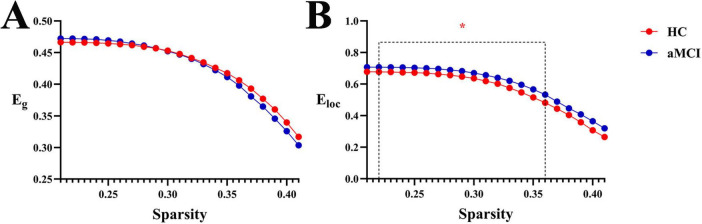
Trend graphs of intergroup comparison of network efficiency in WM structural networks across sparsity (0.21∼0.41). *Indicates *P* < 0.05 (statistically significant); **(A)** line graph of intergroup comparison of E_g_ across sparsity; **(B)** line graph of intergroup comparison of E_loc_, across sparsity; E_g_, global efficiency; E_loc_, local efficiency.

**FIGURE 10:**
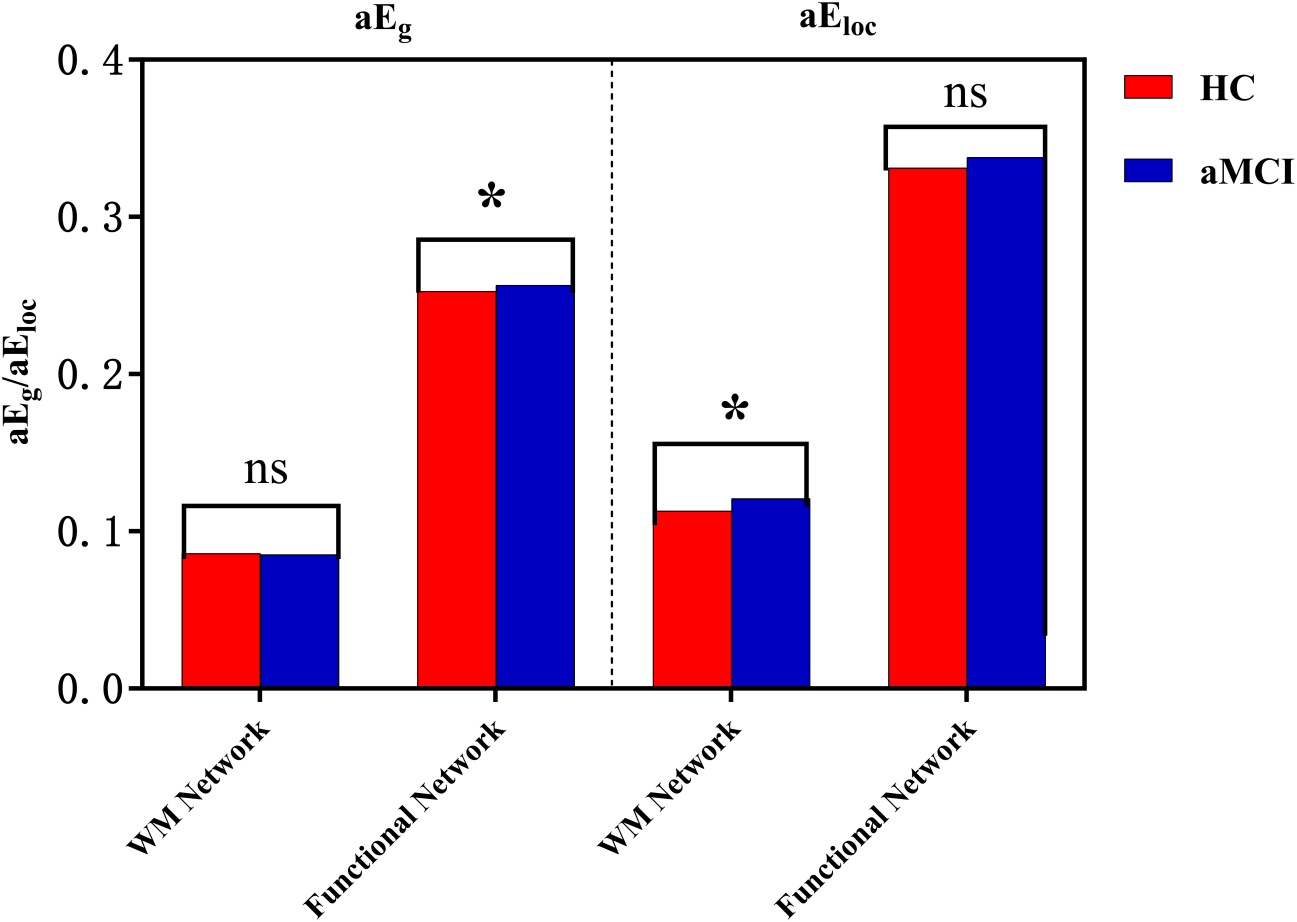
Intergroup comparison of aE_g_ and aE_loc_. *Indicates statistical significance at *P* < 0.05 (independent-samples *t*-test); ns denotes non-significant results. Red, HC group; blue, aMCI group. Covariates including age, gender, education years, MMSE and MoCA scores were controlled where applicable.

In the sparsity range of 0.21∼0.41, intergroup differences in the E_*g*_ index were not statistically significant (*P* > 0.05). However, in the sparsity ranges of 0.22∼0.36, intergroup differences in the E_*loc*_ index were statistically significant (*P* < 0.05). Meanwhile, intergroup comparison of aE_*loc*_ showed statistically significant differences (*P* < 0.05), as shown in Figures 9, 10.

**(2) Functional network**

**FIGURE 11:**
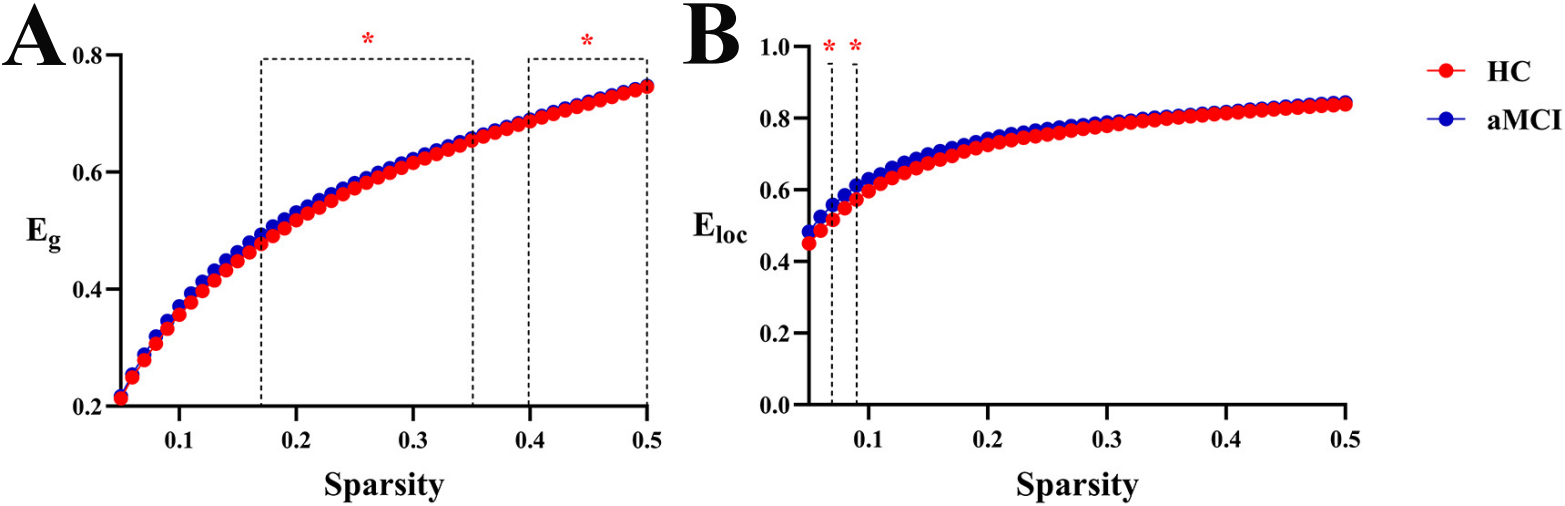
Trend graphs of intergroup comparison of network efficiency in functional networks across sparsity (0.05∼0.5). *Indicates *P* < 0.05 (statistically significant); **(A)** line graph of intergroup comparison of Eg across sparsity; **(B)** line graph of intergroup comparison of E_loc_, across sparsity; E_g_, global efficiency; E_loc_, local efficiency.

Within the sparsity range of 0.17∼0.35 and 0.4∼0.5, there was statistically significant difference in the E_*g*_ index between groups (*P* < 0.05). In the sparsity range of 0.07 and 0.09, the intergroup difference in the E_*loc*_ index was statistically significant (*P* < 0.05). Meanwhile, the intergroup comparison of aE_*g*_ showed a statistically significant difference (*P* < 0.05), as shown in Figure 10, 11.

#### 3.2.4 Assortativity

**(1) WM structural network**

**FIGURE 12:**
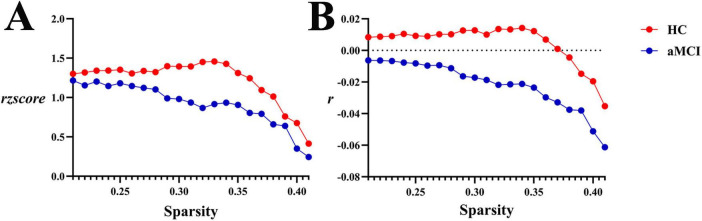
Trend graphs of intergroup comparison of Assortativity in WM structural networks across sparsity (0.21∼0.41). **(A)** Line graph of intergroup comparison of normalized assortativity coefficient *rzscore* across sparsity; **(B)** line graph of intergroup comparison of assortativity coefficient *r* across sparsity; *r*: assortativity coefficient, the AUC of the assortativity of a network for each subject; *rzscore*: normalized assortativity coefficient, the AUC of the z-score of the assortativity of a network determined for each subject by subtracting the average assortativity across random networks and then dividing it by the standard deviation of the assortativity of random networks. The same definitions apply hereinafter.

**FIGURE 13:**
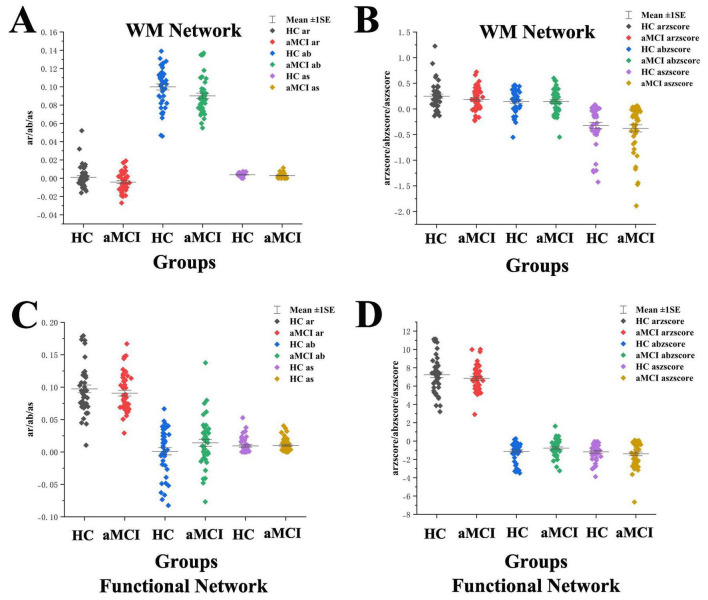
Intergroup comparison of assortativity, hierarchy and synchronization. **(A)** Intergroup comparison of ar, ab and as in WM network. **(B)** Intergroup comparison of arzcore, abzcore and aszcore in WM network. **(C)** Intergroup comparison of ar, ab and as in functional network. **(D)** Intergroup comparison of arzcore, abzcore and aszcore in functional network. Mean ± SE: Mean ± Standard Error.

Within the sparsity range of 0.21∼0.41, there were no statistically significant differences in assortativity coefficients (*r* and *rzscore*) between the two groups (*P* > 0.05). However, in the range of 0.21∼0.37, the HC group showed *r* > 0, indicating an assortative structure in the entire WM structural network, while the aMCI group exhibited a disassortative structure, as shown in Figures 12, 13.

**(2) Functional network**

**FIGURE 14:**
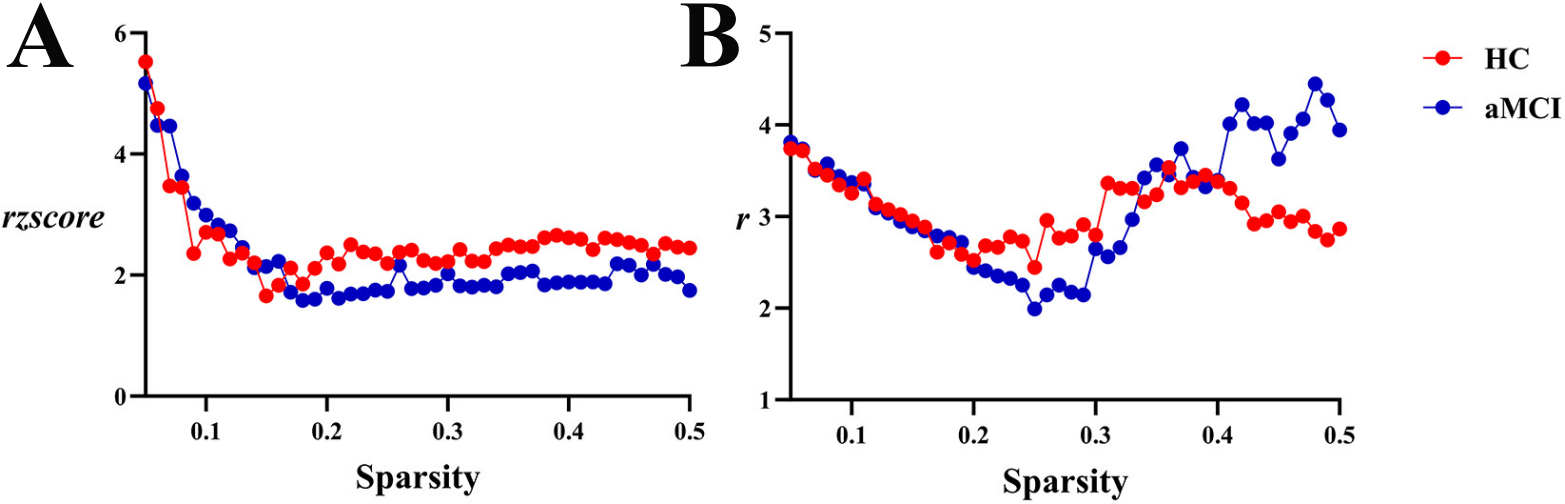
Trend graphs of intergroup comparison of assortativity in functional networks across sparsity (0.05∼0.5). **(A)** Line graph of intergroup comparison of normalized assortativity coefficient *rzscore* across sparsity; **(B)** line graph of intergroup comparison of assortativity coefficient *r* across sparsity.

Within the sparsity range of 0.05∼0.5, there were no statistically significant differences in assortativity coefficients (*r* and *rzscore*) between the two groups (*P* > 0.05). However, in this range, both HC and aMCI groups showed *r* > 0, indicating an assortative structure in the entire functional network, as shown in Figures 13, 14.

#### 3.2.5 Hierarchy

**(1) WM structural network**

**FIGURE 15:**
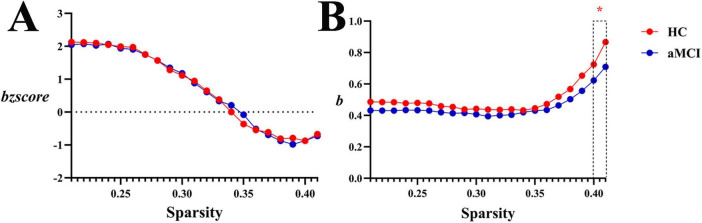
Trend graphs of intergroup comparison of hierarchy in WM structural network across sparsity (0.21∼0.41). **(A)** Line graph of intergroup comparison of normalized hierarchical index *bzscore* across sparsity; **(B)** line graph of intergroup comparison of hierarchical index *b* across sparsity; *b*: hierarchical index, the AUC of the hierarchy of a network for each subject; *bzscore*: The AUC of the z-score of the hierarchy of a network for each subject by subtracting the average hierarchy across random networks and then dividing it by the standard deviation of the hierarchy of random networks. The same definitions apply hereinafter. *Indicating statistical significance, *P* < 0.05 (independent-samples *t*-test).

Within the sparsity ranges of 0.4∼0.41, intergroup difference in *b* were statistically significant (*P* < 0.05). Across the sparsity range of 0.21∼0.41, no significant differences were observed in normalized *bzscore* values between groups (*P* > 0.05), as shown in Figures 15, 13.

**(2) Functional network**

**FIGURE 16:**
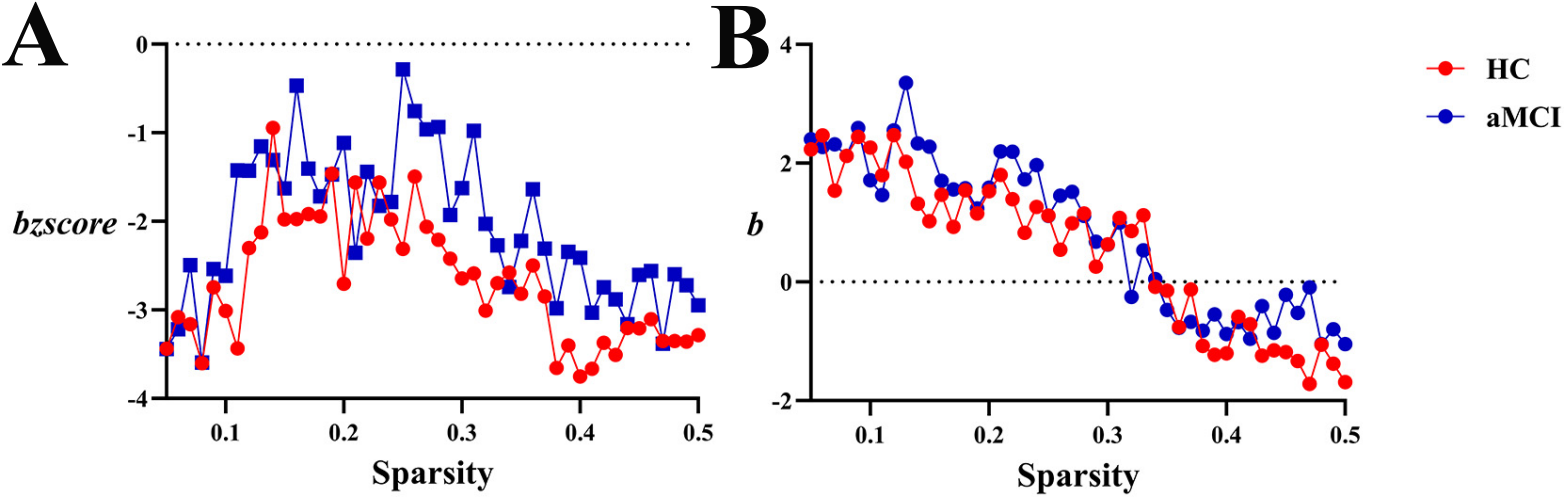
Trend graphs of intergroup comparison of Hierarchy in functional network across sparsity (0.05∼0.5). **(A)** Line graph of intergroup comparison of normalized hierarchical index *bzscore* across sparsity; **(B)** line graph of intergroup comparison of hierarchical index *b* across sparsity.

Within the sparsity ranges of 0.05∼0.5, intergroup differences in hierarchical index *b* and normalized *bzscore* values were no statistically significant (*P* > 0.05). No significant differences were observed in *ab* or *abzscore*, as shown in Figures 16, 13.

#### 3.2.6 Synchronization

**(1) WM structural network**

**FIGURE 17:**
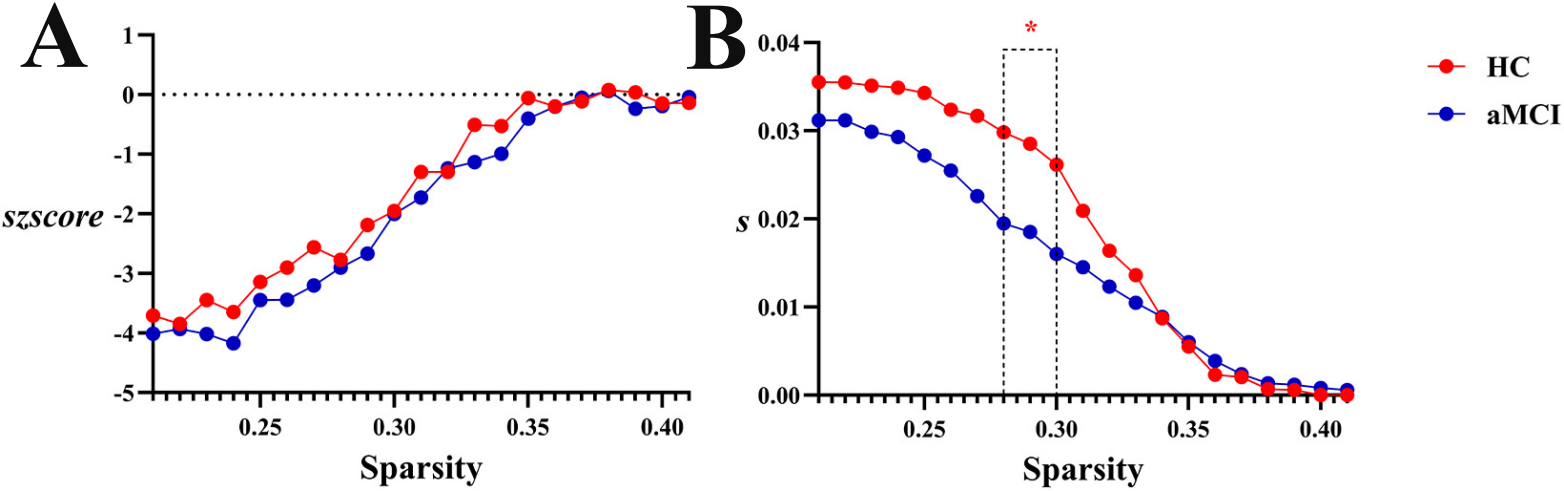
Trend graphs of intergroup comparison of Synchronization in WM structural network across sparsity (0.21∼0.41). **(A)** Line graph of intergroup comparison of normalized global white matter connection strength *szscore* across sparsity; **(B)** line graph of intergroup comparison of global white matter connection strength *s* across sparsity; *s*: global white matter connection strength, The AUC of the synchronization of a network for each subject; *szscore*: normalized global white matter connection strength, the AUC of the z-score of the synchronization of a network determined for each subject by subtracting the average synchronization across random networks and then dividing it by the standard deviation of the synchronization of random networks. *Indicating statistical significance, *P* < 0.05 (independent-samples *t*-test).

Within the sparsity range of 0.21∼0.41, intergroup differences in global white matter connection strength normalized *szscore* values were no statistically significant. However, within the sparsity range of 0.28∼0.30, intergroup differences in global white matter connection strength *s* values were no statistically significant (*P* < 0.05). No significant differences were observed in *as* or *aszscore*, as shown in Figures 13, 17.

**(2) Functional network**

**FIGURE 18:**
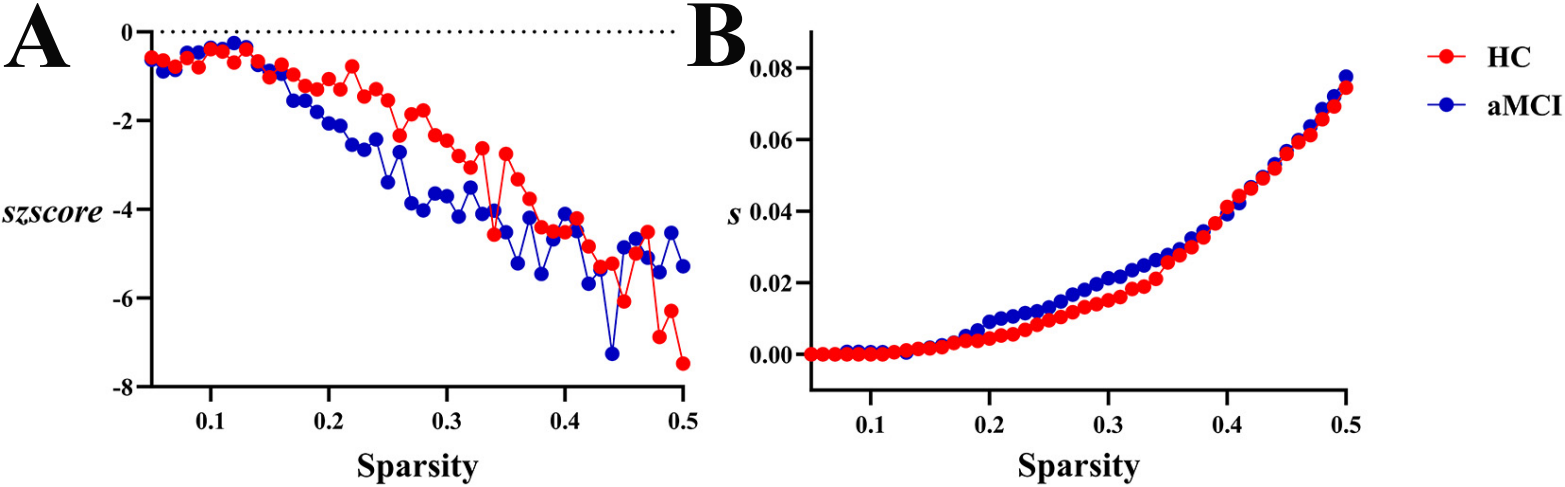
Trend graphs of intergroup comparison of Synchronization in functional network across sparsity (0.05∼0.5). **(A)** Line graph of intergroup comparison of normalized global functional synchrony *szscore* across sparsity; **(B)** line graph of intergroup comparison of global functional synchrony *s* across sparsity.

Within the sparsity range of 0.05∼0.5, there were no statistically significant differences in global functional synchrony *s* or normalized *szscore* between groups. No significant differences were observed in *as* or *aszscore*, as shown in Figures 13, 18.

## 4 Discussion

This study systematically revealed abnormal changes in global topological properties in patients with aMCI through parallel analyses of white matter structural networks and resting-state functional networks. Rather than examining individual metrics in isolation, our study integrated a comprehensive set of global topological measures—including small-world, rich-club, network efficiency, assortativity, hierarchy, and synchronization—enabling a multidimensional characterization of network disruptions in aMCI across both “local-to-global” and “structure-to-function” dimensions, ultimately uncovering a distinct impairment profile. All results were corrected for multiple comparisons using the Bonferroni method and statistically controlled for covariates such as age, sex, years of education, MMSE, and MoCA scores. Our findings indicate that the structural and functional networks in aMCI patients exhibit both interrelated and distinct impairment patterns: structural networks showed reduced global integration efficiency, whereas functional networks demonstrated signs of compensatory reorganization and abnormally elevated local efficiency. This “structure-function dissociation” not only delineates a core characteristic of network abnormalities in aMCI but also provides an objective basis for understanding its neuropathological mechanisms from an integrated structure-function perspective. It should be noted that the number of nodes differed between the white matter structural and functional networks, reflecting modality-specific technical requirements. Nevertheless, employing distinct node definition strategies allowed us to derive reliable and informative network features from both structural and functional dimensions. These two approaches complemented each other, jointly illuminating the complex organizational principles of brain networks. Furthermore, to evaluate the adequacy of our sample size, a *post hoc* statistical power analysis was conducted using G*Power 3.1.9.2. With the current sample size, an effect size of 0.5, and a significance level of 0.05, the achieved statistical power was 0.75. Although this value is slightly below the conventional threshold of 0.80—suggesting that interpretations of non-significant comparisons (i.e., negative results) should be made with caution—it is important to emphasize that all reported significant results (*p* < 0.05) underwent rigorous statistical control, thereby supporting the scientific reliability of these positive findings.

### 4.1 Small-world

This study confirms that both aMCI patients and HC exhibit typical small-world properties in their white matter structural networks. This architecture enables efficient within-module information integration via short-range fibers and facilitates between-module communication through long-range pathways such as commissural and association fibers, playing a crucial role in maintaining the optimal balance between processing efficiency and system robustness (Zhang et al., 2015; Bai et al., 2012). Beyond confirming this fundamental organization, our findings reveal specific topological alterations in aMCI patients. Specifically, the aMCI group demonstrated an overall higher C_*p*_ than the HC group, with significant between-group differences observed in specific sparsity ranges (Figure 1). This result aligns with the findings of Shu et al. (2012) and suggests the presence of a “compensatory enhancement of local clustering” in aMCI brain networks. This implies that when long-distance white matter connections (e.g., corpus callosum, superior longitudinal fasciculus) are compromised, leading to reduced global integration efficiency, the brain network attempts to compensate by strengthening short-range connections within local regions (e.g., within the default mode network) as a passive compensatory mechanism. However, this compensation appears inefficient and potentially detrimental (Tao et al., 2017; Chen et al., 2020; Luo et al., 2019). We further observed that as sparsity increased, C_*p*_, γ, and σ showed a gradual decreasing trend in both groups. This indicates that during network pruning, the brain’s structural network—whether in health or disease—selectively retains functionally significant connections that support local collaborative efficiency to maintain basic information processing capacity. Moreover, the aMCI group exhibited a steeper increase in L_*p*_ with rising sparsity compared to the HC group, indicating accelerated deterioration of global information transfer efficiency and intensified network “fragmentation”. A plausible explanation is that while excessive local clustering aims to compensate, it may instead form “information islands,” thereby impeding cross-module integration and ultimately prolonging L_*p*_. In summary, the evolution of brain networks in aMCI can be understood as a “compensation-damage vicious cycle”: impairment in long-range connections triggers a compensatory increase in local C_*p*_, while excessive local clustering, in turn, exacerbates global integration deficits. These two processes interact and reinforce each other, collectively driving the progression of cognitive decline.

In the investigation of small-world properties within functional networks, we observed that the C_*p*_ exhibited a trend opposite to that of the white matter structural network—namely, C_*p*_ increased with rising sparsity. This finding provides direct evidence for the “structure-function dissociation” between white matter architecture and functional networks. Further analysis revealed that, despite this dissociation, the overall trends in small-world properties of functional networks were largely consistent between the HC and aMCI groups. This suggests that, regardless of health status or aMCI condition, functional networks adhere to a common small-world architectural principle: as sparsity increases, they prioritize the preservation of key functional connections to maintain the fundamental framework of local functional clustering and global functional integration. From a neurobiological perspective, this indicates that in the early stage of neurodegenerative pathology such as aMCI, brain networks can still employ such passive compensatory mechanisms to preserve basic topological attributes, albeit with inherent fragility and inefficiency. As a core metric of global integration efficiency in functional networks, L_*p*_ demonstrated a significant correlation with episodic memory (Xue et al., 2020). In this study, L_*p*_ showed significant between-group differences within specific sparsity ranges, with the aMCI group exhibiting abnormally lowered L_*p*_ values. Conventionally, due to impaired white matter structural connections, L_*p*_ in aMCI patients would be expected to increase (Shu et al., 2012). The L_*p*_ reduction observed here may stem from an over-reinforcement of local functional connectivity in the functional network, leading to an abnormal shortening of global pathways. It must be emphasized that this low L_*p*_ does not signify truly efficient integration but rather serves as a direct manifestation of topological disorganization in the functional network. Collectively, these findings not only align with previous studies but also refine the characterization of abnormal small-world attributes in the brain networks of aMCI patients. Based on these results, future research could focus on these significantly altered metrics—such as C_*p*_ and L_*p*_—to investigate their dynamic changes across different levels of memory impairment or disease stages in aMCI, thereby providing more precise imaging-based insights into the mechanisms of cognitive decline.

### 4.2 Rich-club

Given the absence of existing literature on the Rich-club characteristics of brain networks in aMCI patients, conducting research in this area holds significant innovativeness. Understanding the organizational patterns of Rich-club structures in aMCI patients and their roles in disease progression—such as whether nodal redistribution occurs and how such changes impact network information transmission efficiency—can shed light on the abnormal organization and function of high-order nodes in aMCI brain networks. Research has established that the human structural brain network contains a highly conserved “rich-club” organization—a core hub comprising densely interconnected nodes with high connectivity. Although metabolically costly to maintain, this architecture provides significant topological advantages for global information integration and efficient communication. Evidence suggests that these central connections remain relatively stable during cognitive decline and have been identified as a central backbone in cross-species studies (e.g., in primates), underscoring their pivotal role in whole-brain communication (van den Heuvel and Sporns, 2011; Collin et al., 2014; Senden et al., 2014; Daianu et al., 2015; Schroeter et al., 2015; Harriger et al., 2012).

In this study, calculations of Rich-club indices revealed nodal redistribution of aMCI patients compared to HC groups. In the study of WM structural networks, when the node degree *K* ranged from 3 to 15, the high-degree nodes in the HC group’s WM structural network exhibited a significant Rich-club phenomenon. In contrast, the aMCI group only showed the Rich-club phenomenon when *K* was 6∼15, with its starting node degree delayed by 3 units compared to the HC group. This suggests an abnormal topological threshold in the structural Rich-club organization pattern of aMCI patients. This may be attributed to damage to long-range white matter association fibers in aMCI patients, which leads to the loss of structural connections in low-degree nodes—these nodes thus fail to meet the topological properties of high-degree nodes. Only nodes with more connections can retain sufficient structural links to form the Rich-club phenomenon. Additionally, the normalized Rich-club coefficient of the aMCI group was lower than that of the HC group, indicating reduced structural connection tightness between high-degree nodes and impaired structural connection integrity of the core hub network. In contrast, patients with frontotemporal glioma exhibit significantly increased structural connectivity among rich-club nodes (Liu et al., 2020), suggesting that rich-club organization varies across populations depending on distinct underlying neural mechanisms.

In the functional network study, the HC group showed a stable Rich-club phenomenon when *K* was 2-15, while the aMCI group only exhibited this phenomenon when *K* was 3-15, with a higher starting degree than the HC group. This reveals an abnormal topological threshold also exists in the functional Rich-club organization pattern of aMCI patients. Since functional networks rely on neural activity synchronization rather than physical structural connections, the aMCI group can still form the Rich-club at a relatively low *K* (i.e., *K* = 3), though the threshold is higher than that of the HC group (*K* = 2). This reflects an elevated activity screening threshold for functional core nodes, where a relatively higher number of connections is required to compensate for insufficient functional coupling caused by structural damage. Notably, within a specific degree range, the normalized Rich-club coefficient of the aMCI group was higher than that of the HC group, manifesting as tighter functional connections between high-degree nodes. This essentially represents the functional network compensating for the difficulty in Rich-club formation (caused by elevated topological thresholds) by enhancing the connection synchronization strength of core nodes. However, this compensation may exacerbate the local “information silo” effect, leading to reduced global integration efficiency. These findings reveal abnormal Rich-club organizational patterns in aMCI brain networks, though the underlying neural mechanisms require further exploration. Preliminary speculation suggests that nodal redistribution may relate to neurodegeneration and atrophy in specific brain regions, altering their network importance. Noteworthy limitations include a small sample size and lack of longitudinal tracking. Future studies could expand sample scales, integrate long-term follow-up data, and clarify the dynamic relationship between Rich-club changes and aMCI progression to inform precision intervention strategies.

### 4.3 Network efficiency

Brain network efficiency, as one of the most commonly used and core evaluation indices, is widely applied in the research fields of structural and functional networks. Numerous studies have confirmed that the high efficiency of white matter structural networks supports the efficient operation of functional networks, which is considered the infrastructure for efficient communication in functional networks. In particular, high global efficiency is positively correlated with higher levels of cognitive function. In studies of various neuropsychiatric diseases, researchers have generally observed significant decreases in global or local efficiency of brain networks, suggesting that impaired information transmission in brain networks may be one of the core pathological mechanisms of neuropsychiatric diseases.

In this study, we compared brain network efficiency between aMCI patients and HC groups. The results showed that compared with the HC group, the E_*loc*_ of the WM structural network in aMCI patients was significantly higher, and the E_*loc*_ curve of the aMCI group was higher than that of the HC group across the entire sparsity range. There are two explanations for this phenomenon: first, pathological reorganization—due to damage to major long-range white matter fiber tracts, the brain network is divided into multiple relatively independent and overly dense local modules. Connections within the modules are relatively preserved or even strengthened, resulting in a forced isolated state at the cost of global communication. Second, compensatory response—to cope with early damage, the brain attempts to compensate for the deficiency in global network efficiency by enhancing the internal efficiency of local regions. In addition, the results revealed that when the network was streamlined to retain only the core connections, the E_*g*_ of the aMCI group was lower than that of the HC group under high sparsity. This indicates that substantial damage has occurred to the most critical core Rich-club connections in the structural network of aMCI patients, which is perfectly consistent with the previously observed result of reduced Rich-club coefficient in the aMCI structural network. At low sparsity, the abundant short- and medium-range connections may partially mask or compensate for the damage to core connections, leading to no significant difference in E_*g*_ between the aMCI and HC groups.

At the functional network level, aMCI patients exhibited an advantage in global efficiency, while the change in E_*loc*_ was not significant—this stand in sharp contrast to the structural network (Figures 9–11). This structural-functional dissociation strongly suggests that the aMCI brain undergoes large-scale functional reorganization despite impaired structural connections, which represents a form of compensatory reorganization. The WM structural network of aMCI patients is damaged, particularly the Rich-club hub connections responsible for long-range communication. To address the disruption of structural connections, the brain’s functional network activates a compensatory mechanism, manifested as increased E_g_. This functional compensation can be detected by graph theory analysis (Behfar et al., 2020). It may barely maintain basic cognitive functions for a certain period but cannot support the rapid and accurate information exchange required for high-level cognitive activities (e.g., episodic memory). Consequently, patients still exhibit obvious cognitive impairments, and this compensation may also accelerate the progression of the disease.

### 4.4 Assortativity

Assortativity of brain networks, a key indicator for measuring nodal connection preferences, is crucial for maintaining network stability. It enhances network robustness by protecting hub nodes against random attacks, and the Rich-club phenomenon exemplifies strong assortativity. Notably, human brain structural networks typically exhibit disassortative characteristics: high-degree nodes tend to connect with low-degree peripheral nodes. This unique connection pattern acts as an efficient information transmission channel, enabling rapid convergence of local information to hub nodes for whole-brain information integration. In this study, the Assortativity index of white matter structural networks showed significant intergroup differences: the HC group exhibited *r* > 0 within specific sparsity ranges, indicating assortative connection patterns, which may reflect the stability of information transmission through homogeneous nodal connections in healthy brain networks. In contrast, the aMCI group showed *r* < 0 (disassortative), exhibiting disassortative characteristics. This implies that under pathological conditions, the brain structural network adopts a connection pattern where high-degree nodes tend to connect with low-degree nodes—representing a reorganization of connection strategy from the “high-degree nodes preferentially connecting to high-degree nodes” pattern in healthy brains to a “dominantly high-low connection” pattern. Additionally, in specific sparsity ranges, the HC group showed a higher *rzscore*, indicating that the deviation of its network’s assortative characteristics from random networks was significantly greater than the deviation of the aMCI group’s disassortative characteristics from random networks. This suggests an inherent difference in the topological connection patterns of white matter structural networks between the two groups. Such changes in structural patterns may be associated with brain network functional disturbances during the aMCI stage, providing new evidence for research on the mechanisms of aMCI brain networks. Intriguingly, both groups showed assortativity (*r* > 0) in functional networks, implying that aMCI patients may partially compensate for structural damage by maintaining assortative connections, though the effectiveness of this compensation requires further validation.

### 4.5 Hierarchy

Network hierarchy analysis showed *b* > 0 in both HC and aMCI white matter structural networks (Figure 15), indicating enhanced hierarchy. The *b* remained stable at 0.5, indicating that the hierarchical organization of the HC group’s brain structural network was relatively steady. When sparsity increased beyond 0.38, *b* rose rapidly—this implies that the healthy brain network proactively strengthens its hierarchical structure as connections become sparser, which may serve as an “efficient simplification” strategy to preserve key hierarchical regulation. In contrast, the *b* value of the aMCI group was consistently lower than that of the HC group: it remained flatter at low sparsity, and although it also increased in the later stage, the magnitude of this increase was weaker than that in the HC group. This suggests that the hierarchical organization ability of the aMCI patients’ brain structural network is impaired. The disease state may disrupt the “proactivity” of hierarchical construction; even as connections are simplified, the aMCI brain cannot strengthen the hierarchy at the WM structural level as effectively as the healthy brain, reflecting a decline in network regulatory efficiency. Literature suggests that increased b values in healthy older adults reflect adaptive reorganization during brain aging—with age-related degradation of white matter myelination and axonal integrity (especially long-range connections), the brain optimizes information processing pathways through hierarchical structuring. Whether the hierarchical changes in aMCI patients are driven solely by aging or by combined disease-specific damage and compensation requires longitudinal data for further exploration. In conclusion, as sparsity increased, the structural network of the HC group could proactively strengthen its hierarchy. In contrast, the structural network of the aMCI group exhibited weak hierarchical construction and was prone to disorganization. Notably, the functional network of aMCI patients showed increased hierarchy, which is inferred to reflect compensatory hierarchical enhancement.

### 4.6 Synchronization

Synchronization analysis revealed critical evidence of functional impairment in aMCI brain networks: significantly decreased s values in white matter structural networks directly reflected reduced fiber connection density, indicating impaired functional synchrony. In the HC group, the *s* remained stably at a high level when sparsity ranged from 0.21 to 0.3. This indicates that the healthy WM network can maintain strong synchronization and efficient inter-regional collaboration when connections are relatively dense. As sparsity increased to 0.35–0.5, *s* decreased rapidly but still retained a certain degree of resilience. In contrast, the *s* value of the aMCI group was consistently lower than that of the HC group. This suggests that the synchronization of the WM network in aMCI patients is inherently weaker—once sparsity increases, the efficiency of inter-regional collaborative synchronization decreases. Meanwhile, analysis of the global synchronization strength of the functional network showed that as sparsity increased, functional synchronization in both the HC and aMCI groups increased continuously, though with tiny differences. Overall, the aMCI group exhibited stronger synchronization; however, this synchronization was more disorganized compared to that of random networks. This reflects the dual impairment of the functional network by the disease—specifically, impairment in both its ability to “streamline collaboration” and “enhance synchronization”. This finding aligns with Assortativity and hierarchy changes, outlining a pathological trajectory of “structural connection degradation—connection pattern remodeling—functional synchrony impairment” in aMCI patients. Future research could integrate multimodal data to deeply analyze the causal relationships among changes in different network properties, providing theoretical evidence for elucidating the pathogenesis of aMCI and developing targeted intervention strategies.

## 5 Conclusion

This study simultaneously employed DTI and fMRI techniques to construct brain networks from structural and functional perspectives, and comprehensively analyzed topological property indices including small-world, Rich-club, network efficiency, assortativity, hierarchy, and synchronization. Such a multimodal and multi-index research approach is relatively comprehensive and systematic, which is rare in existing aMCI studies. Within the same cohort, we observed that both the white matter structural and functional networks in patients with aMCI undergo significant topological reorganization, yet exhibit distinct—even opposite—abnormal patterns. This reveals a underlying “structure-function dissociation” mechanism: the structural network shows impairment of its core backbone, weakened global integration, and sparser connectivity, whereas the functional network undergoes compensatory reorganization with abnormally elevated local efficiency, resulting in a more localized, tightly connected, yet increasingly rigid functional architecture.

## 6 Limitations

Although this study clearly delineates topological disparities in brain networks between aMCI patients and healthy controls, it provides a more precise basis for early disease diagnosis and investigation into pathological mechanisms. However, this study has certain limitations. For instance, it did not distinguish between more specific aMCI subtypes (including amnestic single-domain and multi-domain subtypes). Existing studies have indicated significant differences in pathological progression trajectories and clinical prognoses among different subtypes, yet systematic exploration of whether there are specific patterns of brain network abnormalities in these subtypes is still lacking. This may prevent the current study from fully revealing the subtype-specific heterogeneity of brain network damage in aMCI. Stratified analysis of different subtypes or disease course stages of aMCI patients may uncover more valuable information in future research. Xue et al. (2024) found that there were significant differences in small-world properties, Rich-club organization, and other aspects of functional networks among different MCI subtypes. Similarly, Khodadadi Arpanahi et al. (2025) also used graph-theoretical analysis to conduct cross-sectional and longitudinal studies on functional networks across various stages of AD progression (including different MCI subtypes), aiming to provide biomarkers for disease progression. Additionally, this study only analyzed data from patients in a single hospital, resulting in limited sample sources and a small sample size. Future research could make full use of globally recognized datasets, focus on the interrelationships among these indices for integrated analysis, and continuously explore methods for network construction, index calculation, and statistical analysis to improve the reliability, comparability, and interpretability of results. A further limitation lies in the inconsistent number of nodes between the structural and functional networks, which precludes direct quantitative comparison of their topological metrics. Future studies could develop a unified network reconstruction framework or conduct multimodal network analysis within a consistent atlas space.

## Data Availability

The datasets presented in this study can be found in online repositories. The names of the repository/repositories and accession number(s) can be found in the article/supplementary material.
